# End-stage kidney disease due to haemolytic uraemic syndrome – outcomes in 241 consecutive ANZDATA registry cases

**DOI:** 10.1186/1471-2369-13-164

**Published:** 2012-12-03

**Authors:** Wen Tang, Janaki Mohandas, Stephen P McDonald, Carmel M Hawley, Sunil V Badve, Neil Boudville, Fiona G Brown, Philip A Clayton, Kathryn J Wiggins, Kym M Bannister, Scott B Campbell, David W Johnson

**Affiliations:** 1 ANZDATA Registry, Adelaide, Australia; 2 Division of Nephrology, Peking University Third Hospital, Beijing, China; 3 Department of Nephrology, University of Queensland at Princess Alexandra Hospital, Brisbane, Australia; 4 Department of Nephrology and Transplantation services, University of Adelaide at Central Northern Adelaide Renal and Transplantation Services, Adelaide, Australia; 5 School of Medicine and Pharmacology, Sir Charles Gairdner Hospital Unit, The University of Western Australia, Perth, Australia; 6 Department of Nephrology, Monash Medical Centre, Melbourne, VIC, Australia; 7 Department of Renal Medicine, Royal Prince Alfred Hospital, Sydney, Australia; 8 School of Public Health, University of Sydney, Sydney, Australia; 9 Department of Nephrology, Royal Melbourne Hospital, Parkville, VIC, Australia; 10 Department of Renal Medicine,Princess Alexandra Hospital, Level 2, Ambulatory Renal and Transplant Services Building, Ipswich Road, Woolloongabba, Brisbane, Qld, 4102, Australia

**Keywords:** Haemolytic uraemic syndrome, Kidney Failure, Chronic, Outcomes, Renal function recovery, Renal transplantation, Thrombotic microangiopathy

## Abstract

**Background:**

The aim of this study was to investigate the characteristics and outcomes of patients receiving renal replacement therapy for end-stage kidney disease (ESKD) secondary to haemolytic uraemic syndrome (HUS).

**Methods:**

The study included all patients with ESKD who commenced renal replacement therapy in Australia and New Zealand between 15/5/1963 and 31/12/2010, using data from the ANZDATA Registry. HUS ESKD patients were compared with matched controls with an alternative primary renal disease using propensity scores based on age, gender and treatment era.

**Results:**

Of the 58422 patients included in the study, 241 (0.4%) had ESKD secondary to HUS. HUS ESKD was independently associated with younger age, female gender and European race. Compared with matched controls, HUS ESKD was not associated with mortality on renal replacement therapy (adjusted hazard ratio [HR] 1.14, 95% CI 0.87-1.50, p = 0.34) or dialysis (HR 1.34, 95% CI 0.93-1.93, p = 0.12), but did independently predict recovery of renal function (HR 54.01, 95% CI 1.45-11.1, p = 0.008). 130 (54%) HUS patients received 166 renal allografts. Overall renal allograft survival rates were significantly lower for patients with HUS ESKD at 1 year (73% vs 91%), 5 years (62% vs 85%) and 10 years (49% vs 73%). HUS ESKD was an independent predictor of renal allograft failure (HR 2.59, 95% CI 1.70-3.95, p < 0.001). Sixteen (12%) HUS patients experienced failure of 22 renal allografts due to recurrent HUS. HUS ESKD was not independently associated with the risk of death following renal transplantation (HR 0.92, 95% CI 0.35-2.44, p = 0.87).

**Conclusions:**

HUS is an uncommon cause of ESKD, which is associated with comparable patient survival on dialysis, an increased probability of renal function recovery, comparable patient survival post-renal transplant and a heightened risk of renal transplant graft failure compared with matched ESKD controls.

## Background

Haemolytic uraemic syndrome (HUS) is a condition characterised by thrombotic microangiopathy, thrombocytopenia, microangiopathic hemolytic anemia, renal dysfunction, neurologic deficits and sometimes dysfunction of other organs, such as heart, lungs, gastrointestinal tract and pancreas [1,2]. When neurologic dysfunction predominates, the disease is often referred to as thrombotic thrombocytopenic purpura (TTP). HUS is typically caused by Shiga-like toxin-producing bacteria, particularly *E. coli* O157:H7 [3], or non-enteropathic infections (such as with *S. pneumoniae*) [4]. Approximately 10% of HUS cases are not associated with diarrhoea or shiga toxin-producing E. coli and are referred to as atypical HUS [1,5]. This heterogeneous disorder may be either familial or sporadic and can be caused or triggered by complement regulatory protein mutations (50%), complement factor H autoantibodies (6-10%), human immunodeficiency virus infection, autoimmune disorders, cardiovascular surgery, transplantation, disseminated malignancy, pregnancy and certain drugs (including calcineurin inhibitors, muromonab-CD3, valacicylovir, clopidogrel, ticlopidine, bleomycin, gemcitabine and cisplatin) [1,2,6-8]*.*

The worldwide incidence of HUS is reported to be 1–2 cases per 100,000 people per year [1,9]. Progression to end-stage kidney disease (ESKD) occurs in 3% of patients with diarrhoea-associated HUS [10], 50% of atypical HUS cases [1] and 50-80% of familial atypical HUS cases [1]. Previous studies of the predictors, course and outcomes of ESKD patients with HUS have been limited, often restricted to single centre investigations and primarily focused on renal transplantation rather than dialysis [1,2,11,12]. To date, there has not been a comprehensive, multi-centre examination of ESKD secondary to HUS.

The aim of the present study was to investigate the characteristics, treatments and outcomes of all cases of ESKD due to HUS in the Australian and New Zealand dialysis populations, using data from the Australia and New Zealand Dialysis and Transplant (ANZDATA) registry.

## Methods

### Patient population

The cohort study included all patients with ESKD enrolled in the ANZDATA registry, who commenced renal replacement therapy between 15 May 1963 and 31 December 2010. All patients entered into the ANZDATA registry were considered by their treating nephrologists to have ESKD and therefore thought to require long-term renal replacement therapy at the time they were enrolled. The data collected included demographic data, cause of primary renal disease, renal replacement therapy (RRT) dates and modalities, smoking status, body mass index (BMI), late referral (defined as commencement of dialysis within 3 months of referral to a nephrologist), serum creatinine concentration at dialysis commencement, comorbidities (hypertension, chronic lung disease, cardiovascular disease and diabetes mellitus) and outcomes (patient, technique and renal allograft survival). Body mass index (BMI) was calculated from weight/height^2^ and expressed in kg/m^2^. Patients with a primary renal diagnosis of HUS were compared with the remainder of the cohort with an alternative primary renal diagnosis (non-HUS). For each of the RRT, dialysis and renal transplant cohorts, HUS ESKD patients were matched with controls with alternative causes of ESKD by propensity score matching [13]. The propensity score was calculated by using multivariable logistic regression, in which HUS was the outcome variable and age, gender and treatment era were the independent variables. The derived propensity scores were then used to match HUS patients with controls in a 1:1 ratio. Survival analyses were restricted to HUS ESKD patients and matched controls. Ethical approval for the use of registry data was obtained from the Princess Alexandra Hospital Human Research Ethics Committee.

The primary outcomes were patient survival on renal replacement therapy (censored for renal function recovery, loss to follow-up and end of study), patient survival on dialysis (censored for renal function recovery, loss to follow-up, renal transplantation and end of study), time from dialysis commencement to recovery of dialysis-independent renal function (censored for death, loss to follow-up, renal transplantation and end of study), renal transplant patient survival (censored for allograft failure, loss to follow-up and end of study) and renal allograft survival (censored for death, loss to follow-up and end of study). Recovery of dialysis-independent renal function was considered to have occurred if the treating renal unit had recorded that the patient had recovered renal function and completed dialysis therapy. The onset of recovery was defined as the date of the last dialysis treatment.

### Statistical analysis

Results were expressed as frequencies and percentages for categorical variables, mean ± standard deviation for continuous normally distributed variables, and median [interquartile range; IQR] for continuous variables that were not normally distributed. Dichotomous and categorical data were compared using chi-square tests. Continuous normally distributed data were compared using two tailed unpaired t-tests. Continuous non-normally distributed data were compared using Mann–Whitney tests. The independent predictors of HUS ESKD were assessed by multivariate logistic regression analysis. Time to event analyses were evaluated by Kaplan Meier and multivariate Cox proportional hazards survival analyses. The covariates included in the model for the entire cohort were age, gender, racial origin, ESKD cause (HUS or non-HUS), and dialysis era (as well as donor type for renal transplant analyses). In light of the possibility of informative censoring due to differential transplantation rates and renal function recovery rates between patients with and without HUS, multivariate competing-risks regression was also performed for dialysis patient survival analyses [14-16]. A supplementary, fully adjusted analysis was also conducted using a contemporary cohort (1996–2010), in whom data were available on BMI, smoking status, history of chronic lung disease, cerebrovascular disease, ischaemic heart disease, diabetes mellitus, peripheral vascular disease and late referral. Data were analysed using the software package PASW Statistics for Windows release 18.0 (SPSS Inc., North Sydney, Australia) and Stata/SE version 12.0 (StataCorp. CollegeStation, TX). P values less than 0.05 were considered statistically significant.

## Results

### Patient characteristics

Between 15 May 1963 and 31 December 2010, 58422 individuals started RRT for ESKD. Of these, 241 (0.4%) had ESKD secondary to HUS, whilst 58181 (99.6%) had ESKD due to other causes. The baseline characteristics of the two groups before and after matching are displayed in Table 1. Using multivariable logistic regression analysis, ESKD secondary to HUS was significantly and independently associated with younger age (p < 0.001), female gender (p < 0.001) and later dialysis era compared with other forms of ESKD (Table 2). A lower probability of HUS ESKD was observed in patients with Asian racial origin (p = 0.03), Aboriginal and Torres Strait Islander (p = 0.001) or Maori and Pacific Islander racial origin (p < 0.001).

**Table 1:** Characteristics of all patients with ESKD secondary to HUS or other causes in Australia and New Zealand 1963–2010, before and after matching for age, gender and RRT era

	**Before matching**			**After matching**	
**Characteristic**	**HUS ESKD (n = 241)**	**Other ESKD (n = 58181)**	**P value**	**Other ESKD Control (n = 241)**	**P value**
Age	33.1 ± 21.8	54.2 ± 17.3	<0.001	33.1 ± 21.7	1.00
Age category			<0.001		1.00
0-9 years	43 (18%)	529 (1%)		43 (18%)	
10-19 years	28 (12%)	1607 (3%)		28 (12%)	
20-29 years	46 (19%)	3978 (7%)		46 (19%)	
30-39 years	32 (13%)	5772 (10%)		32 (13%)	
40-49 years	31 (13%)	9122 (16%)		31 (13%)	
50-59 years	25 (10%)	12339 (21%)		25 (10%)	
60-69 years	21 (9%)	12866 (22%)		21 (9%)	
70-79 years	13 (5%)	9741 (17%)		13 (5%)	
80+ years	2 (1%)	2327 (4%)		2 (1%)	
Male gender	90 (37%)	33913 (58%)	<0.001	90 (37%)	1.00
Racial origin			<0.001		<0.001
European	217 (90%)	45368 (78%)		170 (71%)	
ATSI	4 (2%)	3484 (6%)		22 (9%)	
MPI	8 (3%)	5106 (9%)		24 (10%)	
Asian	6 (3%)	2478 (4%)		9 (4%)	
Other	6 (3%)	1745 (3%)		16 (7%)	
RRT era			0.17		1.00
1963-1975	13 (5%)	2821 (5%)		12 (5%)	
1976-1985	36 (15%)	6505 (11%)		36 (15%)	
1986-1995	44 (18%)	12119 (21%)		45 (19%)	
1996-2000	50 (21%)	9811 (17%)		49 (20%)	
2001-2005	44 (18%)	12357 (21%)		45 (19%)	
2006-2010	54 (22%)	14568 (25%)		54 (22%)	
Ever smoked			<0.001		0.85
Current	28 (12%)	6138 (11%)		31 (13%)	
Former	42 (17%)	17713 (30%)		48 (20%)	
Never	130 (54%)	22398 (39%)		122 (51%)	
Missing	41 (17%)	11932 (21%)		40 (17%)	
Hypertension			<0.001		0.98
Yes	89 (37%)	24464 (42%)		87 (36%)	
No	37 (15%)	4519 (8%)		38 (16%)	
Missing	115 (48%)	29198 (50%)		116 (48%)	
Diabetes mellitus			<0.001		<0.001
Yes	8 (3%)	17874 (31%)		51 (21%)	
No	201 (83%)	31796 (55%)		163 (68%)	
Missing	32 (13%)	8511 (15%)		27 (11%)	
Chronic lung disease			0.009		0.47
Yes	16 (7%)	7139 (12%)		22 (9%)	
No	190 (79%)	41125 (71%)		190 (79%)	
Missing	35 (15%)	9917 (17%)		29 (12%)	
Coronary artery disease			<0.001		0.13
Yes	22 (9%)	17613(30%)		36 (15%)	
No	184 (76%)	30768 (53%)		176 (7%)	
Missing	35 (15%)	9800 (17%)		29 (12%)	
Peripheral vascular disease			<0.001		0.04
Yes	15 (6%)	11440 (20%)		31 (13%)	
No	191 (79%)	36823 (63%)		181 (75%)	
Missing	35 (15%)	9918 (17%)		29 (12%)	
Cerebrovascular disease			0.01		0.38
Yes	14 (6%)	6540 (11%)		9 (4%)	
No	192 (80%)	41742 (72%)		204 (84%)	
Missing	35 (15%)	9899 (17%)		29 (12%)	
BMI (kg/m^2^)	22.7 ± 6.2	26.6 ± 6.3	<0.001	24.5 ± 7.7	0.02
Late referral			<0.001		<0.001
Yes	88 (37%)	9304 (16%)		34 (14%)	
No	86 (36%)	33773 (58%)		156 (65%)	
Missing	67 (28%)	15104 (26%)		51 (21%)	
Renal Biopsy			<0.001		<0.001
Yes	89 (37%)	16019 (28%)		85 (35%)	
No	61 (25%)	29135 (50%)		117 (49%)	
Miss	91 (38%)	13027 (22%)		39 (16%)	
First RRT			0.46		0.02
Haemodialysis	163 (68%)	39135 (67%)		134 (56%)	
Peritoneal dialysis	69 (29%)	17570 (30%)		92 (38%)	
Renal Transplant	9 (4%)	1476 (3%)		15 (6%)	
RRT duration (years)	4.7 [1.6-11.8]	3.8 [1.6-8.3]	0.03	6.4 [2.3-14.3]	0.03

Abbreviations: *ATSI* Aboriginal and Torres Strait Islander, *BMI* Body mass index, *MPI* Maori and Pacific Islander, *RRT* Renal replacement therapy.

**Table 2:** Multivariable logistic regression analysis of predictors of ESKD due to HUS, as opposed to other causes of ESKD, in both the entire cohort (limited covariate adjustment) and a contemporary cohort in which complete data were available on comorbidities

**Characteristic**	**Entire cohort (1963–2010) (n = 58442)**	**Contemporary cohort (1996–2010) (n = 36884)**
Age (per decade)	0.54 (0.50-0.57)	0.63 (0.57-0.69)
Male gender	0.40 (0.31-0.52)	0.40 (0.28-0.57)
Racial origin		
European	Reference	Reference
ATSI	0.18 (0.07-0.47)	0.26 (0.08-0.85)
MPI	0.26 (0.13-0.52)	0.33 (0.14-0.77)
Asian	0.40 (0.18-0.92)	0.37 (0.15-0.91)
Other	NS	0.42 (0.15-1.15)
RRT era		NS
1963–1975	0.36 (0.19-0.66)	
1976–1985	0.55 (0.36-0.85)	
1986–1995	0.53 (0.36- 0.80)	
1996–2000	1.03 (0.69-1.51)	
2001–2005	0.86 (0.57-1.28)	
2006-2010	Reference	
Ever smoked	NA	NS
Diabetes mellitus	NA	0.13 (0.06-0.31)
Chronic lung disease	NA	NS
Coronary artery disease	NA	NS
Peripheral vascular disease	NA	NS
Cerebrovascular disease	NA	NS
BMI (kg/m^2^)	NA	NS
Late referral	NA	3.72 (2.65-5.22)

Abbreviations: *ATSI* Aboriginal and Torres Strait Islander, *BMI* Body mass index, *MPI* Maori and Pacific Islander, *NA* Not analysed due to incomplete data prior to 1996, *NS* Not statistically significant, *RRT* Renal replacement therapy

Results are reported as adjusted odds ratio (95% CI).

In a supplementary analysis using a more contemporary cohort (1996–2010) in which complete data were available on comorbidities (n = 36884 including 148 patients with HUS ESKD), HUS ESKD was significantly and independently associated with younger age (p < 0.001), female gender (p < 0.001), late referral (p < 0.001) and lower probability of diabetes mellitus (p < 0.001), Asian racial origin (p = 0.03), Aboriginal and Torres Strait Islander racial origin (p = 0.03) or Maori and Pacific Islander racial origin (p = 0.11).

### Patient survival on renal replacement therapy

Overall, the survival of patients with HUS ESKD on RRT (median 11.7 years, 95% CI 2.72-20.6 years) was comparable to that of matched controls (median 16.6 years, 95% CI 11.4-21.7 years; log rank score 0.83, p = 0.36). Using multivariable Cox proportional hazards model analysis, HUS was not independently associated with mortality on RRT (adjusted hazard ratio [HR] 1.14, 95% CI 0.87-1.50, p = 0.34) in the entire cohort after adjusting for racial origin (p = 0.22), and in the contemporary cohort (1996–2010) (HR 1.11, 95% CI 0.68-1.80, p = 0.68) after adjusting for racial origin (p = 0.55), chronic lung disease (HR 2.08 95% CI 1.17-3.69, p = 0.01), cerebrovascular disease (HR 2.03 95% CI 1.04-3.93, p = 0.04), ischaemic heart disease (HR 2.13 95% CI 1.28-3.57, p = 0.004), diabetes mellitus (HR 1.94 95% CI 1.06-3.55, p = 0.03), peripheral vascular disease (p = 0.49), BMI (p = 0.52), late referral (p = 0.10) and smoking status (p = 0.54).

### Patient survival on dialysis

**Figure 1:**
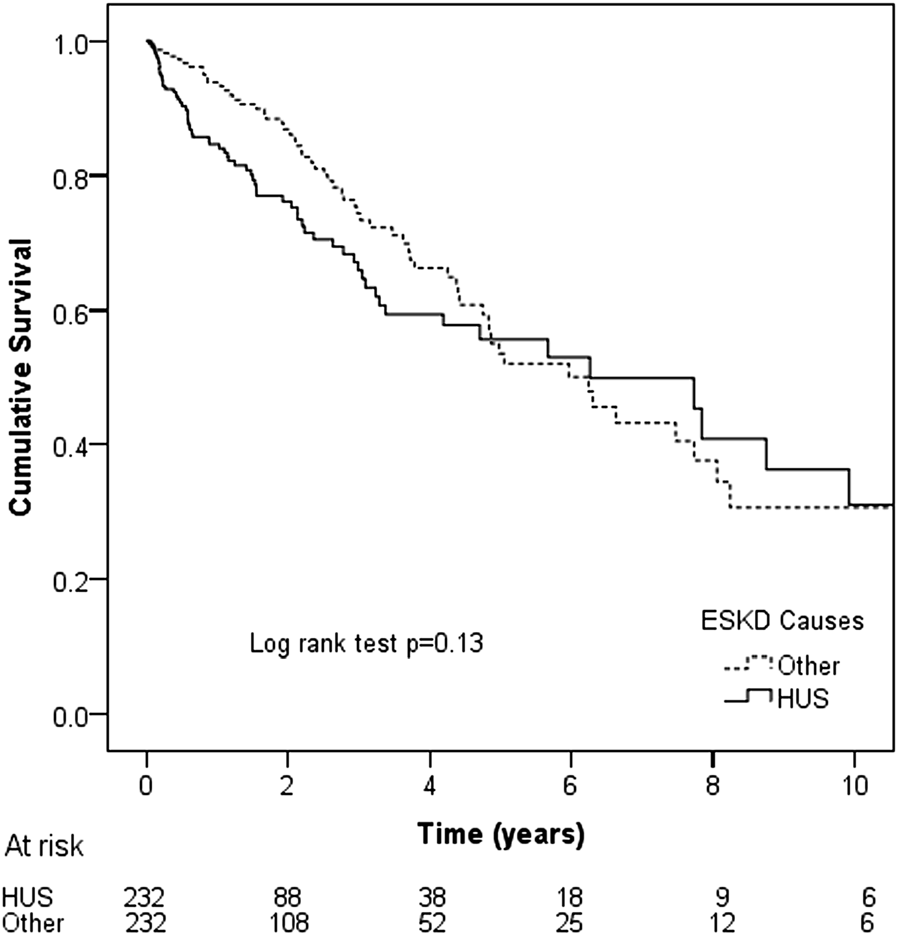
Kaplan-Meier survival curves for HUS dialysis ESKD and matched control dialysis patients with other causes of ESKD in Australian and New Zealand dialysis patients 1963–2010

Death occurred in 68 (29%) HUS ESKD dialysis patients and 62 (27%) matched dialysis controls (p = 0.54). The causes of death were cardiac (31% vs 40%, respectively), withdrawal from dialysis (21% vs 23%), infections (21% vs 10%), vascular (10% vs 10%), malignancy (3% vs 5%) and other (15% vs 13%) (overall p = 0.58). Median patient survival on dialysis was comparable between HUS ESKD (6.26 years, 95% CI 3.79-8.73 years) and matched controls (6.24 years, 95% CI 4.59-7.89 years, log rank score 2.28, p = 0.13) (Figure 1). Respective survival rates in the two groups were 85% vs 94% at 1 year, 76% vs 87% at 2 years and 56% vs 52% at 5 years. Using multivariable Cox proportional hazards model analysis, HUS was not a predictor of mortality on dialysis in the entire cohort (HR 1.35, 95% CI 0.93-1.96,p = 0.12) after adjusting for racial origin (p = 0.84), or in the contemporary cohort (HR 0.91, 95% CI 0.53-1.56, p = 0.73) after adjusting for racial origin (p = 0.67), chronic lung disease (HR 2.24 95% CI 1.23-4.08, p = 0.008), cerebrovascular disease (p = 0.25), ischaemic heart disease (p = 0.32), diabetes mellitus (p = 0.23), peripheral vascular disease (p = 0.16), BMI (p = 0.10), late referral (p = 0.15) and smoking status (p = 0.26). HUS was not an independent predictor of mortality when renal transplantation and renal function recovery were treated as competing events in competing-risk analysis (HR 1.34, 95% CI 0.93-1.93, p = 0.12) after adjusting for racial origin.

When only patients with HUS dialysis were considered, death on dialysis was predicted by older age (HR per decade 1.31, 95%CI 1.16-1.49, p < 0.001) and initiation of dialysis in a later era (1996–2000 HR 0.34, 95% CI 0.12-0.97, p = 0.045; 2001–2005 HR 0.22, 95% CI 0.07-0.67, p = 0.008; 2006–2010 HR 0.21, 95% CI 0.07-0.65, p = 0.007; 1963–1975 reference), after adjusting for gender (p = 0.42) and racial origin (p = 0.21).

### Recovery of renal function

Recovery of dialysis-independent renal function occurred in 21 (9%) HUS ESKD patients and 5 (2%) matched controls (p = 0.001). Time to renal recovery was significantly shorter in HUS ESKD (log rank score 11.2, p = 0.001). Using multivariable Cox proportional hazards model analysis, HUS was a significant independent predictor of renal function recovery in the entire cohort (HR 4.01, 95% CI 1.45-11.1, p = 0.008) after adjusting for racial origin. Six (29%) HUS patients and 2 (40%) matched controls never returned to renal replacement therapy by the end of the study (31 December 2010). One (5%) HUS patient and 1 (20%) control died directly after recovery of renal function. One (5%) HUS patient received a renal transplant after 1.06 years. Thirteen (62%) HUS patients and 2 (40%) controls returned to dialysis after median periods of 0.94 years (interquartile range 0.19-5.54 years) and 0.19 years (interquartile range 0.06-0.31 years), respectively (p = 0.17).

### Renal transplant graft survival

A total of 130 (54%) patients with HUS ESKD received 166 renal allografts during the study period compared with 19549 (34%) non-HUS ESKD patients who received 22773 renal allografts (p < 0.001). Excluding patients undergoing pre-emptive renal transplantation (HUS ESKD n = 9, non-HUS ESKD n = 1476), the median time from dialysis commencement to first renal transplant was comparable for HUS patients (1.40 years, 95% interquartile range 0.80-2.57) and non-HUS patients (1.44 years, 95% CI 0.64-2.9, p = 0.9). The baseline characteristics of these patients before and after matching are shown in Table 3. Overall, the features of the two groups were similar, except that HUS patients were younger and less likely to have chronic lung disease, diabetes mellitus, coronary heart disease, peripheral vascular disease, cerebral vascular disease or hypertension. They were more likely to be female, non-smoker, referred late to a renal unit, transplanted after 1996 and have received an allograft from a living donor.

**Table 3:** Characteristics of all patients with ESKD secondary to HUS or other causes in Australia and New Zealand who underwent renal transplantation during the period 1963–2010, before and after matching for age, gender and transplant era

	**Before matching**			**After matching**	
**Characteristic**	**HUS ESKD (n = 130)**	**Other ESKD (n = 19549)**	**P value**	**Other ESKD Control (n = 130)**	**P value**
Age	25.2 ± 17.1	41.9 ± 14.8	<0.001	25.2 ± 17.0	0.98
Male gender	52 (40%)	11704 (60%)	0.001	52 (40%)	1.00
Racial origin			0.1		0.01
European	118 (91%)	16738 (86%)		104 (80%)	
ATSI	1 (1%)	489 (3%)		3 (2%)	
MPI	4 (3%)	812 (4%)		11 (9%)	
Asian	1 (1%)	925 (5%)		9 (7%)	
Other	6 (5%)	585 (3%)		3 (2%)	
Transplant era			0.01		1.00
1963–1975	4(3%)	1939 (10%)		4 (3%)	
1976–1985	23 (18%)	3448 (18%)		22 (17%)	
1986–1995	22 (17%)	4595 (24%)		22 (17%)	
1996–2000	26 (20%)	2667 (14%)		27 (21%)	
2001–2005	24 (19%)	3150 (16%)		24 (19%)	
2006-2009	31 (24%)	3750 (19%)		31 (24%)	
Ever smoked			<0.001		0.37
Current	10 (8%)	1560 (8%)		4 (3%)	
Former	14 (11%)	3668 (19%)		15 (12%)	
Never	82 (63%)	7788 (40%)		90 (69%)	
Missing	24 (19%)	6533 (33%)		21 (16%)	
Hypertension			0.009		0.35
Yes	54 (42%)	8562 (44%)		43 (33%)	
No	23 (18%)	1898 (10%)		24 (19%)	
Missing	53 (41%)	9089 (47%)		63 (49%)	
Diabetes mellitus			<0.001		0.007
Yes	1 (1%)	2098 (11%)		11 (9%)	
No	113 (87%)	13435 (69%)		109 (84%)	
Missing	16 (12%)	4016 (21%)		10 (8%)	
Chronic lung disease			0.007		0.28
Yes	1 (1%)	581 (3%)		1 (1%)	
No	111 (85%)	14333 (73%)		119 (92%)	
Missing	18 (14%)	4637 (24%)		10 (8%)	
Coronary artery disease			0.002		0.25
Yes	4 (3%)	1201 (6%)		3 (2%)	
No	108 (83%)	13484(69%)		117 (90%)	
Missing	18 (14%)	4864 (25%)		10 (8%)	
Peripheral vascular disease			0.003		0.28
Yes	1 (1%)	703 (4%)		1 (1%)	
No	111 (85%)	14137 (72%)		119 (92%)	
Missing	18 (14%)	4709 (24%)		10 (8%)	
Cerebrovascular disease			0.025		0.03
Yes	4 (3%)	391 (2%)		0 (0%)	
No	108 (83%)	14525(74%)		120 (92%)	
Missing	18 (14%)	4633 (24%)		10 (8%)	
BMI (kg/m^2^)			<0.001		0.86
Late referral	22.0 ± 5.9	24.7 ± 5.1	<0.001	21.8 ± 5.9	0.001
Yes	35 (27%)	1742 (9%)		15 (12%)	
No	56 (43%)	10294 (53%)		84 (65%)	
Missing	39 (30%)	7513 (38%)		31 (24%)	
Donor type			<0.001		1.00
Deceased	72 (55%)	14540 (74%)		72 (55%)	
Living	58 (45%)	5009 (26%)		58 (45%)	
Subsequent grafts			0.06		0.44
2	32 (19%)	2718 (12%)		22 (14%)	
3	3 (2%)	440 (2%)		4 (3%)	
4	1 (0%)	61 (0%)		0 (0%)	
5	0 (0%)	5 (0%)		0 (0%)	

Abbreviations: *ATSI* Aboriginal and Torres Strait Islander, *BMI* Body mass index, *MPI* Maori and Pacific Islander, *RRT* Renal replacement therapy.

**Figure 2:**
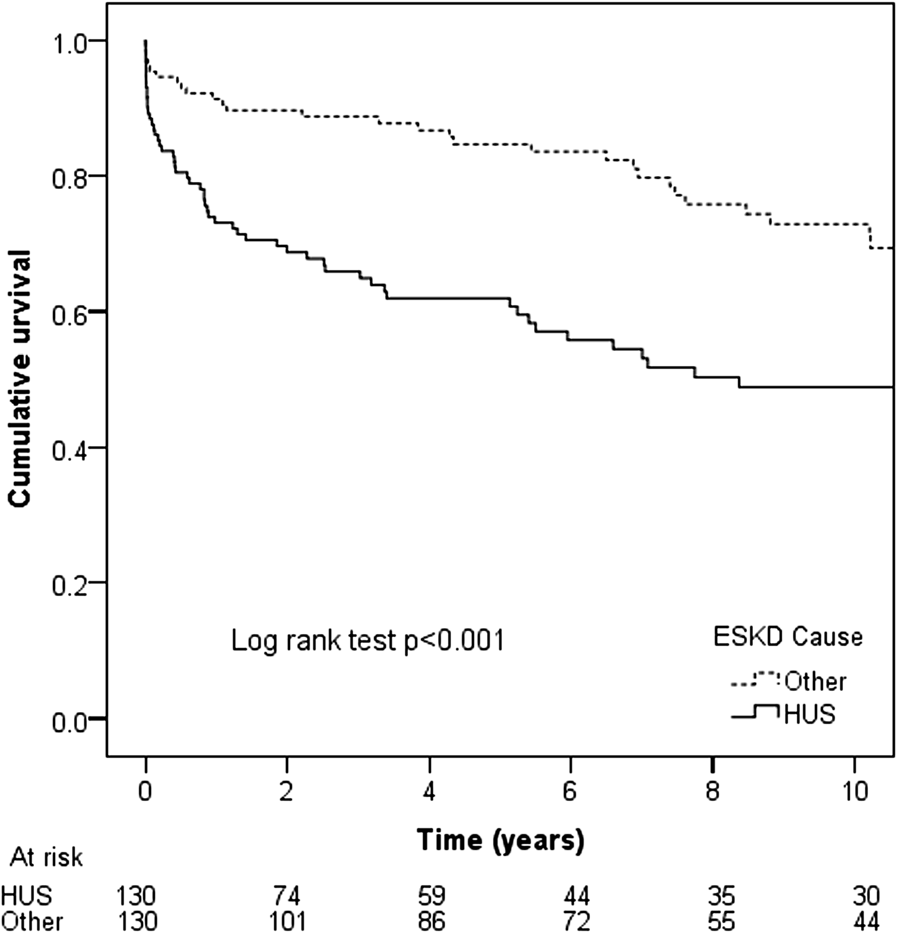
Kaplan-Meier death-censored first graft survival curves for HUS ESKD and matched control patients with other causes of ESKD undergoing renal transplantation in Australian and New Zealand between 1963 and 2010

Renal allograft survival rates in patients with HUS ESKD were generally inferior to those matched ESKD controls, regardless of graft number (all, first or subsequent), donor type (living or deceased) and transplant era. Mean death-censored first graft survivals for HUS and matched ESKD controls were 12.0 years (95% CI 9.38-14.5 years; 10 year survival 49%) and 24.6 years (95% CI 20.7-28.4 years, 10 year survival 73%, p < 0.001; Figure 2), respectively. The respective values for first grafts from deceased donors were 11.2 years (95% CI 7.69-14.7 years, 10 year survival 41%) and 21.6 years (95% CI 17.7-25.5, 10 year survival 71%) (p < 0.001), whilst those for first grafts from living donors were 12.3 years (95% CI 9.04-15.7 years, 10 year survival 59%) and 26.8 years (95% CI 20.9-30.6 years, 10 year survival 76%) (p = 0.02). Using multivariable Cox proportional hazards analysis, HUS was an independent predictor of graft failure (HR 2.65, 95% CI 1.73-4.05, p < 0.001), after adjusting for racial origin (p = 0.84) and donor type (living versus deceased; p = 0.21).

When only HUS patients were considered, graft failure was independently predicted by renal transplant era (1963–1975 reference; 1976–1985 HR 0.29, 95% CI 0.08-1.09, p = 0.07; 1986–1995 HR 0.24, 95% CI 0.06-0.91, p = 0.04; 1996–2000 HR 0.17, 95% CI 0.04-0.66; 2001–2005 HR 0.25, 95% CI 0.07-0.94, p = 0.04; 2006–2010 HR 0.02 95% CI 0.01-0.21, p < 0.001; overall p = 0.01), after adjusting for age (p = 0.38), gender (p = 0.47), racial origin (p = 0.93) and donor type (p = 0.51).

### HUS recurrence in renal transplants

Sixteen (12%) HUS patients experienced failure of 22 renal allografts due to recurrent HUS (Table 4). The median time between kidney transplantation and loss of graft from recurrent HUS was 0.82 years (interquartile range 0.18-3.10 years). Of the 16 HUS patients who lost their first renal allograft from HUS, 8 underwent a second kidney transplant and 3 (37.5%) lost these grafts from recurrent HUS. Conversely, of the 51 HUS patients who lost their first renal allograft for reasons other than recurrent HUS, 23 underwent a second kidney transplant and 3 (13%) lost these grafts from recurrent HUS.

**Table 4:** Causes of renal allograft failure in all patients with ESKD secondary to HUS or other causes who underwent renal transplantation in Australia and New Zealand during the period 1963–2010

**Characteristic**	**HUS ESKD (n = 87)**	**Other ESKD control (n = 44)**
HUS	22 (25%)	0 (0%)
Hyperacute rejection	3 (3%)	0 (0%)
Acute rejection	13 (15%)	4 (9%)
Chronic allograft nephropathy	27 (31%)	19 (43%)
Renal artery thrombosis	5 (6%)	3 (7%)
Renal vein thrombosis	2 (2%)	1(2%)
Glomerulonephritis	1 (1%)	5 (11%)
Other	14 (16%)	12 (27%)

The differences between the groups were highly statistically significant (p = 0.002).

### Renal transplant patient survival

**Figure 3:**
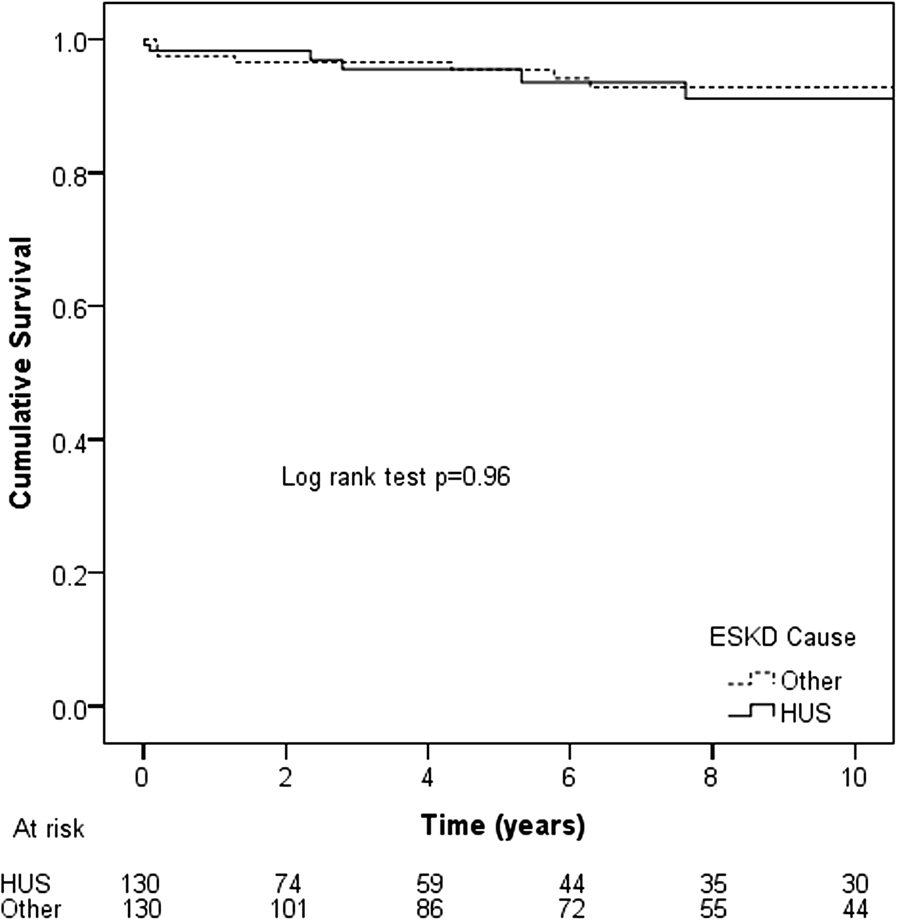
Kaplan-Meier patient survival curves for HUS ESKD and matched control patients with other causes of ESKD undergoing first renal transplantation in Australian and New Zealand between 1963 and 2010

When first renal allografts were considered, the overall survival of HUS patients (10-year survival 91%) was comparable to that of matched ESKD controls (10 year survival 93%, p = 0.96; Figure 3). HUS ESKD was not independently associated with the risk of death following renal transplantation (HR 1.01, 95% CI 0.38-2.69, p = 0.98) after adjusting for donor type (living donor HR 0.16 95% CI 0.04-0.72, p = 0.02) and racial origin (p = 0.96). The causes of death in the HUS (n = 7) and matched ESKD control groups (n = 11) were generally comparable (overall p = 0.21): cardiac (43% vs 9%, respectively), vascular (14% vs 36%), malignancy (29% vs 9%), infection (0% vs 27%) and other (14% vs 18%).

## Discussion

This retrospective, multi-centre, multi-country registry analysis examined the outcomes of 241 patients with HUS ESKD over 46 years. The principal findings were that HUS ESKD was a rare cause of ESKD (0.4% of cases) and was independently associated with younger age, female gender and European racial origin. HUS ESKD was also independently associated with a higher probability of recovery of dialysis-independent renal function and increased risks of renal allograft failure compared with other causes of ESKD.

Very few studies have comprehensively examined the predictors and outcomes of HUS ESKD, particularly in dialysis populations. One previous investigation by the Forum of ESKD Networks in the United States reported 199 cases of HUS ESKD [11]. In keeping with the findings of the present study, the Forum observed a preponderance of young, white, female patients. The respective proportions of patients aged under 20 years, whites and females were 30% vs 33%, 91% vs 88% and 63% vs 71%. These findings are likely explained by the fact that HUS typically affects children and younger adults, particularly females [17,18]. The effect of ethnicity on HUS risk has not been well studied, although African-Caribbean ancestry has been observed as a risk factor [17].

Survival of HUS ESKD patients on dialysis has also not been previously well described. The Forum of ESKD Networks [11] found that the overall survival of HUS patients was 77% at 3 years and somewhat better than that seen in diabetic ESRD controls. However, the authors noted that “this probably reflects in part the lower average age of the patients.” In the present investigation, the 3-year renal replacement therapy survival rates of ESKD patients with HUS and matched controls with other causes of ESKD were 74% and 80%, respectively. Following adjustment for racial origin and comorbidities, patients with HUS ESKD had comparable survival. This also applied to patients treated with dialysis, even after adjusting for the competing risks of renal transplantation and recovery of renal function. The causes of death in the HUS patients were similar to those of other ESKD patients and the limited numbers did not permit useful, multivariable, sub-group analyses.

Although the overall mortality rate of HUS ESKD patients was comparable to that of matched controls, HUS patients were also more likely to experience spontaneous recovery of dialysis-independent renal function. A total of 21 (8.7%) HUS ESKD patients recovered renal function, although 13 (62%) of these patients returned to dialysis after a median period of 0.94 years. There have been previous reports of renal recovery in patients with HUS, even after relatively long periods on dialysis [19-22]. Nissenson et al. [11] observed that 9% of patients with ESKD secondary to HUS were able to discontinue dialysis. Moreover, our group has previously found HUS to be an independent predictor of dialysis-independent renal function recovery in ESKD patients [23,24]. The appreciable incidence of renal recovery suggests that caution should be exercised when listing HUS ESKD patients for renal transplantation within the first year following dialysis commencement.

Although the overall rate of renal transplantation was considerably higher in HUS (54%) than in other causes of ESKD (34%), renal allograft outcomes were significantly worse. Compared with non-HUS patients, HUS patients had significantly inferior overall renal allograft survival rates at 1 year (73% vs 91%, respectively), 5 years (62% vs 85%) and 10 years (49% vs 73%). The one-year first renal allograft survival rates in HUS ESKD patients were 69% for deceased donor transplants and 79% for living donor transplants. These findings are appreciably better than those reported for 78 patients by the International Registry of Recurrent and Familial HUS/TTP (32% and 50%, respectively). Part of the apparent disparity in findings may be potentially explained by HUS population heterogeneity, vintage bias and ascertainment bias (since the International HUS/TTP Registry included data provided by selected, interested global renal units as well as that obtained from literature searches, whereas the ANZDATA Registry included all patients with HUS ESKD who ever received renal replacement therapy in Australia and New Zealand since 1963). For patients who did not experience renal allograft failure, possibly reflecting a less severe form of HUS, their overall survival was superior to their non-HUS counterparts.

HUS recurrence occurred in 12% of HUS patients undergoing renal transplantation after a median period of 0.82 years and accounted for 25 of all renal allograft failures in patients with HUS ESKD. These findings were somewhat lower than those of other investigations reporting recurrence rates of 25-50% [1,2,12], but may have related to HUS population heterogeneity (due to inclusion of patients with typical HUS who had a low risk of post-transplant recurrence), classification bias due to difficulties in distinguishing HUS recurrence from antibody-mediated rejection, and possible ascertainment and reporting bias. In keeping with the findings of other studies [25], living kidney donation was a significant risk factor for HUS recurrence in the renal allograft.

The strengths of this study included its very large sample size and inclusiveness. We included all HUS patients receiving renal replacement therapy in Australia and New Zealand during the study period, such that a variety of centres were included with varying approaches to the treatment of HUS and ESKD. This greatly enhanced the external validity of our findings. These strengths should be balanced against the study’s limitations, which included limited depth of data collection. ANZDATA does not collect important information, such as distinction between typical (diarrhoea-associated) and atypical HUS (which has an important effect on dialysis and transplant outcomes), date of HUS diagnosis, severity of comorbidities, patient compliance, individual unit management protocols (including plasma exchange and eculizumab therapy), laboratory values (such as platelet counts, serum lactate dehydrogenase concentrations and serum ADAMTS13 measurements) and complement regulatory protein genetic mutation and autoantibody results. Even though we adjusted for a large number of patient characteristics, the possibility of residual confounding could not be excluded. In common with other Registries, ANZDATA is a voluntary Registry and there is no external audit of data accuracy, including the diagnosis of HUS.

## Conclusion

In conclusion, HUS is an uncommon cause of ESKD, which is associated with comparable patient survival following dialysis or kidney transplantation, and a significantly increased probability of dialysis-independent renal function recovery. Renal transplantation is a safe and effective therapy for HUS ESKD, although renal allograft survival rates are worse than for patients with other ESKD causes. HUS recurrence occurs in 12% of patients and is the second commonest cause of graft loss in this group after chronic allograft nephropathy. Caution should be exercised in transplanting HUS patients within the first year of dialysis commencement due to the possibility of renal function recovery.

## Competing interests

Professor David Johnson is a consultant for Baxter Healthcare Pty Ltd and has previously received research funds from this company. He has also received speakers’ honoraria and research grants from Fresenius Medical Care and has previously been a consultant to Gambro. He is a current recipient of a Queensland Government Health Research Fellowship. Dr Kym Bannister is a consultant for Baxter Healthcare Pty Ltd. Dr Fiona Brown is a consultant for Baxter and Fresenius and has received travel grants from Amgen and Roche. Dr Stephen McDonald has received speaking honoraria from AMGEN Australia, Fresenius Australia and Solvay Pharmaceuticals and travel grants from AMGEN Australia, Genzyme Australia and Janssen-Cilag. Associate Professor Neil Boudville has previously received research funds from Roche, travel grants from Roche, Amgen and Jansen Cilag, and speaking honoraria from Roche. The remaining authors have no competing financial interests to declare.

## Authors’ contributions

WT conceptualized the idea, analyzed the data and wrote part of the first draft of the manuscript;JM wrote the first draft of the manuscript, interpretation of results and reviewing the drafts of manuscript; SM: contributed to interpretation of data and reviewing the drafts of manuscript; CH and SB: are involved in data analysis, interpretation of results and reviewing the drafts of manuscript; NB, FB, PC, KW, KB and SC: contributed to interpretation of data and reviewing the drafts of manuscript. DJ: is the principal investigator of this study, involved in data analysis and drafting the manuscript. All authors approved the last version of the manuscript.

## Pre-publication history

The pre-publication history for this paper can be accessed here:

http://www.biomedcentral.com/1471-2369/13/164/prepub
